# Linezolid- and Multidrug-Resistant Enterococci in Raw Commercial Dog Food, Europe, 2019–2020

**DOI:** 10.3201/eid2708.204933

**Published:** 2021-08

**Authors:** Ana R. Freitas, Liliana Finisterra, Ana P. Tedim, Bárbara Duarte, Carla Novais, Luísa Peixe, from the ESCMID Study Group on Food- and Water-borne Infections (EFWISG)

**Affiliations:** Instituto Universitário de Ciências da Saúde (IUCS) Departamento de Ciências, Cooperativa de Ensino Superior Politécnico e Universitário (CESPU), CRL, Gandra, Portugal (A.R. Freitas);; UCIBIO, Faculdade de Farmácia, Universidade do Porto, Porto, Portugal (A.R. Freitas, L. Finisterra, B. Duarte, C. Novais, L. Peixe);; Hospital Universitario Rio Hortega/Instituto de Investigación Biomédica de Salamanca, Valladolid/Salamanca, Spain (A.P. Tedim)

**Keywords:** raw dog food, multidrug resistance, enterococci, linezolid resistance, *optrA*, *poxtA*, *cfrD*, ampicillin resistance, cgMLST, bacteria, Portugal, Spain, Europe

## Abstract

We describe enterococci in raw-frozen dog food commercialized in Europe as a source of genes encoding resistance to the antibiotic drug linezolid and of strains and plasmids enriched in antibiotic-resistance and virulence genes in hospitalized patients. Whole-genome sequencing was fundamental to linking isolates from dog food to human cases across Europe.

Raw meat–based diets are increasingly popular for feeding dogs, but the extent of antimicrobial-resistant bacteria in raw dog food is rarely addressed globally (*1*). The Centers for Disease Control and Prevention does not recommend feeding raw diets to pets because of frequent contamination with *Salmonella* and *Listeria* (https://www.cdc.gov/healthypets/publications/pet-food-safety.html), but awareness about this issue is not as evident in Europe. Eating raw meat has been considered a risk factor for carriage of clinically relevant ampicillin-resistant (AmpR) *Enterococcus faecium* and *optrA*-positive linezolid-resistant *E. faecalis* in dogs (*2**,**3*), but data for commercial pet food are not available. We evaluated multidrug-resistant (MDR) *Enterococcus* in raw-frozen dog food commercialized in countries in Europe; we focused on transferable linezolid resistance (LinR) genes because linezolid is a last-resort drug to treat gram-positive infections (*4*).

We purchased 14 raw-frozen dog food samples from the 2 commercially available brands in Portugal in specialized stores (September 2019–January 2020). Brand A (produced in Europe) is available in specialized stores, brand B (produced in the United Kingdom) in specialized stores and online; both are commercialized across different countries in Europe. We enriched samples (25 g) in buffered peptone water (1:10), then in brain–heart infusion broth with or without different antibiotic drugs (ampicillin [16 μg/mL], vancomycin [6 μg/mL], chloramphenicol [16 μg/mL]), and plated them onto Slanetz-Bartley agar with and without the same drug concentrations. We identified isolates with different morphologies per plate by PCR. We performed antibiotic susceptibility testing by disk diffusion using European Committee on Antimicrobial Susceptibility Testing (EUCAST) (*5*) or Clinical and Laboratory Standards Institute (*6*) guidelines. We used broth microdilution for linezolid and Etest for ampicillin. We searched acquired LinR genes (*optrA/poxtA/cfrA-E*) and typed representative isolates by multilocus sequence typing (n = 20; https://www.pubmlst.org) and whole-genome sequencing (LinR *E. faecalis* [n = 6] and AmpR/LinR *E. faecium* [n = 5]) using the Hi Seq 2500 Sequencing System (Illumina, https://www.illumina.com). We deposited assemblies (SPAdes version 3.11.1; https://cab.spbu.ru/software/spades) in GenBank (Bioproject PRJNA663240) and characterized them using in silico tools (http://www.genomicepidemiology.org) and in-house databases *(**7**)*.

All samples carried enterococci resistant to erythromycin, streptomycin, chloramphenicol, and tetracycline; 93% resistant to ampicillin, ciprofloxacin, and quinupristin/dalfopristin; 79% resistant to gentamicin; and 50% resistant to linezolid. We detected acquired LinR genes among 20 MDR isolates from 64% of samples from both brands and with different types of ingredients (Table): *optrA* (4 *E. faecalis*, 1 *E. faecium*), *poxtA* (2 *E. faecium*), *optrA*+*poxtA* (8 *E. faecalis*, 3 *E. faecium*) or *optrA+cfrD* (2 *E. faecalis*). Of those, 15 expressed LinR (MIC = 8 mg/L), whereas 5 were susceptible (MIC = 4 mg/L) (Table).

**Table:** Characterization of *Enterococcus* isolates obtained from raw dog food samples, Porto, Portugal, 2019–2020*

Species	cgMLST†	MLST‡	Sample (brand)§	Antimicrobial drug resistance profile#	Antibiotic resistance genotype	MIC LIN, mg/L	Transfer of LinR genes
*E. faecalis*	CT1206	ST40	Duck (B)	ERY, TET, CHL, LIN	*optrA, fexA, cat, erm(B), Isa(A), tet(M), dfr(G) *	8	–
	CT1207	ST674	Salmon (A)	CIP, ERY, TET, STR, CHL, LIN	*optrA, cfrD, fexA, cat, ant(6)-Ia, aph(3′)-III, erm(B), Isa(A), tet(M), tet(L), dfr(G)*	8	++
	CT1205	ST1008	Turkey (A)¶	ERY, TET, GEN, STR, CHL	*optrA, poxtA, fexB, cat, aac(6')-aph(2”), ant(6)-Ia, ant(9)-Ia, aph(3′)-III, erm(B), lnu(B), Isa(A), Isa(E), tet(M), tet(L), dfr(G)*	4	–
	CT1205	ST1008	Turkey (A)¶	ERY, TET, STR, CHL	*optrA, poxtA, fexB, cat, aac(6')-aph(2”), ant(6)-Ia, ant(9)-Ia, aph(3′)-III, erm(B), lnu(B), Isa(A), Isa(E), tet(M), tet(L), dfr(G)*	4	–
	CT1209	ST1008	Chicken + lamb (A)	ERY, TET, STR, CHL, LIN	*optrA, poxtA, fexB, cat, aac(6')-aph(2”), ant(6)-Ia, ant(9)-Ia, aph(3′)-III, erm(B), lnu(B), Isa(A), Isa(E), tet(M), tet(L), dfr(G)*	8	–
	CT1208	ST1009	Turkey + goose (B)	ERY, CHL, LIN	*optrA, cfrD, fexA, cat, erm(B), Isa(A), dfr(G)*	8	–
*E. faecium*	CT106	ST80	Salmon (A)	AMP (>256 mg/L), CIP, ERY, TET, GEN, STR, QD	*aac(6')-aph(2”), ant(6)-Ia, aph(3′)-III, erm(B), msr(C), tet(M), tet(L), dfr(G)*	ND	NA
	CT284	ST25	Beef (A)	AMP (32 mg/L), CIP, ERY, TET, GEN, STR, QD, CHL	*poxtA, fexB, aac(6')-aph(2”), ant(6)-Ia, ant(9)-Ia, aph(3′)-III, erm(A), erm(B), msr(C), Inu(B), Isa(E), tet(M), tet(L), dfr(G)*	4	–
	CT374	ST264	Beef (A)	AMP (32 mg/L), CIP, TET, STR, QD	*cat, ant(6)-Ia, Inu(G), tet(M), tet(L), dfr(G)*	ND	NA
	CT272	ST1091	Duck (B)	AMP (>256 mg/L), CIP, ERY, TET, STR, QD	*ant(9)-Ia, erm(A), erm(B), msr(C), tet(M), tet(L), dfr(G)*	ND	NA
	CT3399	ST1263	Deer (B)	AMP, ERY, TET, STR, QD, CHL	*poxtA, fexB, cat, ant(6)-Ia, ant(9)-Ia, aph(3′)-III, erm(A), msr(C), Inu(B), Isa(E), tet(L), dfr(G)*	4	+

*AMP, ampicillin; cgMLST, core-genome MLST; CIP, ciprofloxacin; CHL, chloramphenicol; CT, complex type; ERY, erythromycin; GEN, high-level resistance to gentamicin; LIN, linezolid; LinR, linezolid-resistant; MLST, multilocus sequence typing; NA, not applicable; ND, not done: QD, quinupristin/dalfopristin; STR, high-level resistance to streptomycin; ST, sequence type: +, positive (transfer frequency of 10^−8^); ++, positive (transfer frequency of 10^−7^); –, negative. †The *E. faecalis* CT1205-CT1209 and the *E. faecium* CT3399 were identified in this study by submitting them to the cgMLST database (https://www.cgMLST.org) through Ridom SeqSphere^+^ version 7.2 software (https://www.ridom.de/seqsphere). ‡The novel *E. faecalis* ST1008–ST1009 were submitted to the MLST database (https://www.pubmlst.org). §Brand A is produced in Europe; Brand B is produced in the United Kingdom. ¶These 2 samples correspond to 2 different batches and were acquired at different times (October 2019 and January 2020). #QD resistance was tested only against *E. faecium* isolates. Successful transfer of ampicillin resistance is underlined (AMP) and all transconjugants exhibited high values of ampicillin resistance (16–256 mg/L).and.

The *E. faecium* isolates (n = 39) were mostly MDR (70%), expressing resistance to tetracycline (85%), quinupristin/dalfopristin (72%), erythromycin (64%), ciprofloxacin (59%), streptomycin (57%), ampicillin (56%), gentamicin (23%), chloramphenicol (21%), or linezolid (10%). We compared selected dog food AmpR *E. faecium* genomes with 7,660 available GenBank *E. faecium* genomes by complex types (CTs) through core-genome multilocus sequence typing (Ridom SeqSphere^+^ version 7.2, https://www.ridom.de/seqsphere). Those data (Figure) and data from single-nucleotide polymorphisms (Appendix Figure 1) showed different clusters grouping related isolates obtained from dog food and hospitalized patients (sequence type [ST] 80/CT106; ST264/CT374) or from pet food and livestock or wastewaters (ST1091/CT284; ST1263/CT3399) in different countries. Dog food *E. faecium* was enriched in acquired antibiotic-resistant and virulence genes as strains from different sources (Appendix Figure 1). ST80* E. faecium* from brand A was phylogenetically related to other strains from Germany and Netherlands; ST1091 and ST1263 from brand B were phylogenetically related to UK strains (Figure). By filter-mating (*8*), we found that 3 (ST25, ST80, ST1263) of 5 AmpR* E. faecium* isolates transferred a chromosomal genetic platform containing *pbp5* to GE1* E. faecium* strain (Table). Following our previous description of a large transferable *pbp5*-containing platform in a clinical isolate (*8*), we partly identified highly similar genetic platforms carrying different adaptive features including virulence genes (e.g., *sgrA*) in ST80 and ST1263 dog food AmpR* E. faecium* (Appendix Figure 2). ST1263* E. faecium* was able to transfer *poxtA* by conjugation (Table).

**Figure:**
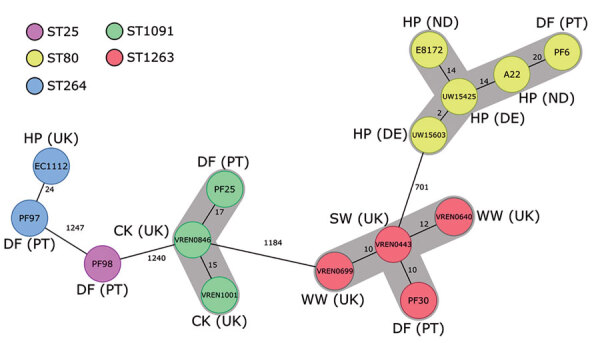
Minimum-spanning tree based on the core-genome multilocus sequence typing (cgMLST) data from *Enterococcus faecium* isolates (n = 15) from different sources in Europe. The tree is based on cgMLST (1,423 genes) analyses made with Ridom SeqSphere^+^ version 7.2 software (https://www.ridom.de/seqsphere). Each circle represents 1 allele profile. The numbers on the connecting lines represent the number of cgMLST allelic differences between 2 isolates. Sequence types are shown in colored circles (see key); numbers in circles are isolate identifications. Gray shading around nodes indicates clusters of closely related isolates (<20). CK, chicken; DE, Denmark; DF, dog food; HP, hospitalized patient; PT, Portugal; ST, sequence type; SW, swine; UK, United Kingdom; WW, wastewater.

The *E. faecalis* isolates (n = 52) recovered were mostly MDR (75%), resistant to chloramphenicol (83%), tetracycline (79%), erythromycin (75%), streptomycin (63%), gentamicin (31%), linezolid (21%), or ciprofloxacin (10%). ST40, ST674, ST1008, and ST1009 sequences corresponded to novel complex types carrying antimicrobial resistance (*aac(6')-aph(2″)/ant(6)-Ia/aph3″-III/erm(B)/tet(M),tet(L),dfr(G)*) and virulence (*ace/gelE/elrA*) genes linked to clinically relevant MDR lineages (Table) (*7**,**9*). ST674 *E. faecalis* carried *optrA* on a pheromone-responsive plasmid (pAPT110) identical to others from non–clonally related *E. faecalis* in hospitalized patients in Spain and China (Appendix Figure 3). Similarly to pAPT110 in this study transferring *optrA* in high rates (Table), pEF10748 (China) is an *optrA* highly transferable plasmid with a complete sex-pheromone response module (*10*).

In conclusion, the diversity and rate of *E. faecium* and *E. faecalis* with linezolid-resistance genes (*optrA/poxtA/cfrD*) we identified were unexpectedly high. Our data suggest that raw dog food could be a sentinel of emerging antimicrobial resistance traits because this type of food may accumulate raw ingredients of different origins, namely from animals associated with intensive farming, adding a new concern to the global health burden of antimicrobial resistance.

Appendix Additional information about drug-resistant enterococci in raw commercial dog food, Europe, 2019–2020.

## References

[1] Davies RH, Lawes JR, Wales AD. Raw diets for dogs and cats: a review, with particular reference to microbiological hazards. J Small Anim Pract. 2019;60:329–39. 10.1111/jsap.13000 31025713 PMC6849757

[2] van den Bunt G, Top J, Hordijk J, de Greeff SC, Mughini-Gras L, Corander J, et al. Intestinal carriage of ampicillin- and vancomycin-resistant *Enterococcus faecium* in humans, dogs and cats in the Netherlands. J Antimicrob Chemother. 2018;73:607–14. 10.1093/jac/dkx455 29294027

[3] Wu Y, Fan R, Wang Y, Lei L, Feßler AT, Wang Z, et al. Analysis of combined resistance to oxazolidinones and phenicols among bacteria from dogs fed with raw meat/vegetables and the respective food items. Sci Rep. 2019;9:15500. 10.1038/s41598-019-51918-y 31664106 PMC6820769

[4] Bender JK, Cattoir V, Hegstad K, Sadowy E, Coque TM, Westh H, et al. Update on prevalence and mechanisms of resistance to linezolid, tigecycline and daptomycin in enterococci in Europe: Towards a common nomenclature. Drug Resist Updat. 2018;40:25–39. 10.1016/j.drup.2018.10.002 30447411

[5] European Committee on Antimicrobial Susceptibility Testing (EUCAST). Breakpoint tables for interpretation of MICs and zone diameters. EUCAST version 10.0; 2020 [cited 2020 Dec 1]. https://www.eucast.org/fileadmin/src/media/PDFs/EUCAST_files/Breakpoint_tables/v_11.0_Breakpoint_Tables.pdf

[6] Clinical and Laboratory Standards Institute. Performance standards for antimicrobial susceptibility testing: twenty-eighth informational supplement M100*.* Annapolis Junction (MD): The Institute; 2018.

[7] Freitas AR, Tedim AP, Novais C, Lanza VF, Peixe L. Comparative genomics of global *optrA*-carrying *Enterococcus faecalis* uncovers a common chromosomal hotspot for *optrA* acquisition within a diversity of core and accessory genomes. Microb Genom. 2020;6:e000350. 10.1099/mgen.0.000350 32149599 PMC7371108

[8] Novais C, Tedim AP, Lanza VF, Freitas AR, Silveira E, Escada R, et al. Co-diversification of *Enterococcus faecium* core genomes and PBP5: evidences of *pbp5* horizontal transfer. Front Microbiol. 2016;7:1581. 10.3389/fmicb.2016.01581 27766095 PMC5053079

[9] Raven KE, Reuter S, Gouliouris T, Reynolds R, Russell JE, Brown NM, et al. Genome-based characterization of hospital-adapted *Enterococcus faecalis* lineages. Nat Microbiol. 2016;1:15033. 10.1038/nmicrobiol.2015.33 27572164

[10] Zou J, Tang Z, Yan J, Liu H, Chen Y, Zhang D, et al. Dissemination of linezolid resistance through sex pheromone plasmid transfer in *Enterococcus faecalis.* Front Microbiol. 2020;11:1185. 10.3389/fmicb.2020.01185 32582110 PMC7288747

