# Supplementary material for: Linezolid- and Multidrug-Resistant Enterococci in Raw Commercial Dog Food, Europe, 2019–2020
Source: Emerg Infect Dis. 2021 Aug;27(8):2221–4. doi: 10.3201/eid2708.204933 (PMC8314808; doi:10.3201/eid2708.204933)
Supplement: Appendix — Additional information about drug-resistant enterococci in raw commercial dog food, Europe, 2019–2020. [file 20-4933-Techapp-s1.pdf]

Article DOI: https://doi.org/10.3201/eid2708.204933

Linezolid- and Multidrug-Resistant Enterococci in Raw Commercial Dog Food, Europe, 2019–2020

Appendix

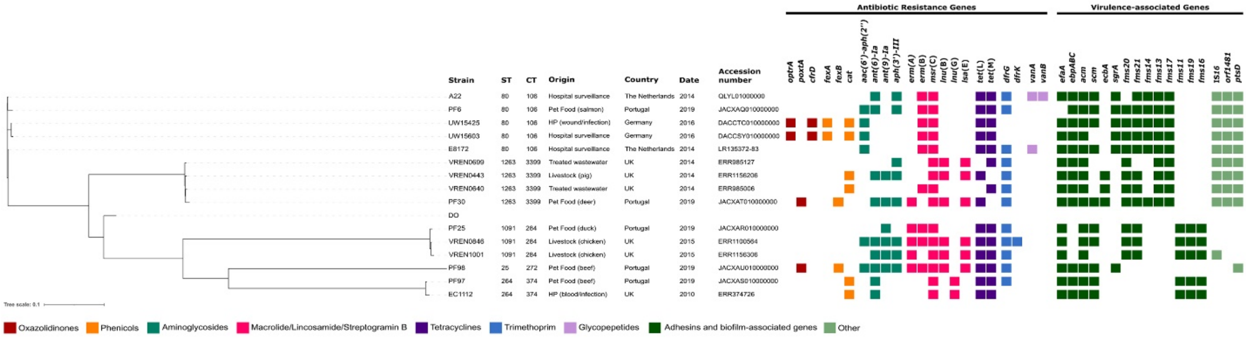

Appendix Figure 1. SNP-based phylogenetic tree comparing *E. faecium* isolates (n = 15) of different sources. SNP alignment was obtained using CSI Phylogeny (https://cge.cbs.dtu.dk/services/CSIPhylogeny) and *E. faecium* DO (GenBank accession no. CP00358) as reference strain. Input parameters were 10x minimum depth and minimum relative depth at SNP positions; SNPs were filtered out if they were called within the vicinity of 300 bp of another SNP (pruning); minimum SNP quality of 30; minimum read mapping quality of 25; minimum Z-score of 1.96. Data were analyzed using FastQC (http://www.bioinformatics.babraham.ac.uk/projects/fastqc) to test the quality of the raw and preprocessed data; SPAdes v.3.11.1 (https://cab.spbu.ru/software/spades) for de novo assembling the paired-end reads; and QUAST (http://quast.bioinf.spbau.ru) for evaluating the quality of genome assembly. Colored cells represent the presence of acquired antimicrobial drug resistance or virulence genes with each color indicating the correspondent family, as indicated in the keys.

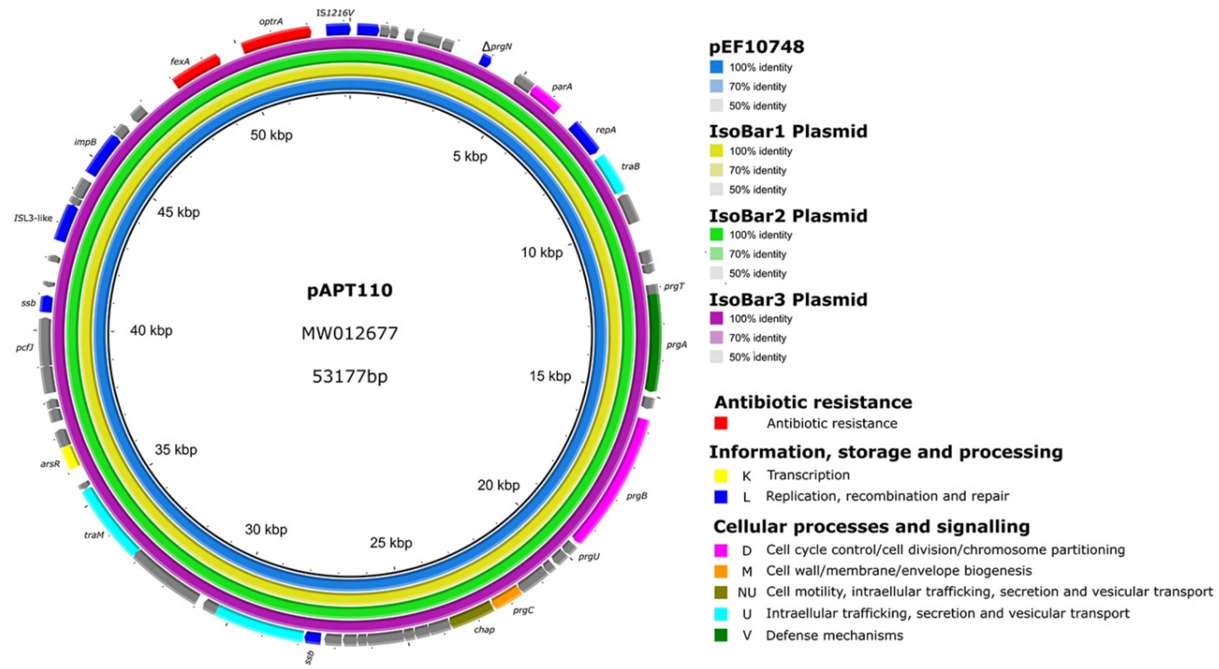

Appendix Figure 2. Representation of partial transferable chromosomal genetic platforms containing *pbp5*. Mapping and annotation (Geneious Prime version 2020.2.2; http://www.geneious.com) of partial transferable *pbp5* chromosomal genetic platforms of selected AmpR-*Efm* PF6/ST80 (68209 bp), PF30/ST1263 (74804 bp) and PF25/ST1091 (13613 bp) were performed using *pbp5*-containing contig of the ampicillin-resistant transconjugant TCGEHPH2 (63575 bp; GenBank accession no. MBRI01000000). The contigs identified were assembled using Vector NTI advance v.11 and the platform annotated using eggNOG-mapper.

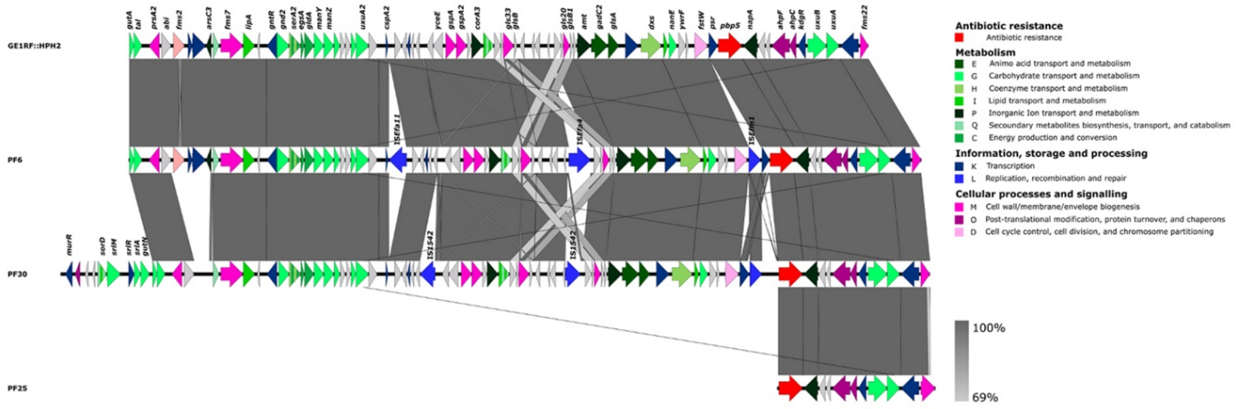

Appendix Figure 3. BLAST Ring Image Generator (BRIG) alignment of 5 *optrA*-carrying plasmids from different isolation sources and geographic regions. The novel *optrA*-carrying plasmid pAPT110 (deposited under GenBank accession no. MW012677) obtained from a raw pet food ST674 *E. faecalis* was annotated using Vector NTI advance v.11 (https://www.thermofisher.com) and eggNOG-mapper

(http://eggnog-mapper.embl.de), and used as a reference plasmid. The outermost circle is an annotation of the reference plasmid and shows the direction of transcriptional open-reading frames. Genes encoding for antibiotic resistance (red), information, storage, and processing (yellow and dark blue), and cellular processes and signaling (pink, orange, greens, and light blue) are indicated. pEF10748 (GenBank accession no. MK993385) was identified in a ST480 *E. faecalis* from a hospitalized patient in China (2015) and Isobar1 (European Nucleotide Archive [ENA] accession no. ERX2067873), Isobar2 (ENA accession no. ERX2067874), and Isobar3 (ENA accession no. ERX2067875) plasmids were recovered from closely related ST585 strains in hospitalized patients in Spain (2016).
